# Changes in intermuscular connectivity during active elbow extension reveal a functional simplification of motor control after stroke

**DOI:** 10.3389/fnins.2022.940907

**Published:** 2022-10-06

**Authors:** Célia Delcamp, Camille Cormier, Alexandre Chalard, David Amarantini, David Gasq

**Affiliations:** ^1^Toulouse NeuroImaging Center, Université de Toulouse, Inserm, UPS, Toulouse, France; ^2^Department of Functional Physiological Explorations, University Hospital of Toulouse, Hôpital de Rangueil, Toulouse, France; ^3^Department of Neurology, University of California, Los Angeles, Los Angeles, CA, United States; ^4^California Rehabilitation Institute, Los Angeles, CA, United States

**Keywords:** brain injuries, common drive theory, electromyography, intermuscular coherence, neuromuscular plasticity

## Abstract

**Background:**

Stroke alters muscle co-activation and notably leads to exaggerated antagonist co-contraction responsible for impaired motor function. However, the mechanisms underlying this exaggerated antagonist co-contraction remain unclear. To fill this gap, the analysis of oscillatory synchronicity in electromyographic signals from synergistic muscles, also called intermuscular coherence, was a relevant tool.

**Objective:**

This study compares functional intermuscular connectivity between muscle pairs of the paretic and non-paretic upper limbs of stroke subjects and the dominant limb of control subjects, concomitantly between two muscle pairs with a different functional role, through an intermuscular coherence analysis.

**Methods:**

Twenty-four chronic stroke subjects and twenty-four healthy control subjects were included. Subjects performed twenty elbow extensions while kinematic data and electromyographic activity of both flexor and extensor elbow muscles were recorded. Intermuscular coherence was analyzed in the beta frequency band compared to the assessment of antagonist co-contraction.

**Results:**

Intermuscular coherence was higher in the stroke subjects’ paretic limbs compared to control subjects. For stroke subjects, the intermuscular coherence of the antagonist-antagonist muscle pair (biceps brachii—brachioradialis) was higher than that of the agonist-antagonist muscle pair (triceps brachii—brachioradialis). For the paretic limb, intermuscular coherence of the antagonist-antagonist muscle pair presented a negative relationship with antagonist co-contraction.

**Conclusion:**

Differences in intermuscular coherence between the paretic limbs of stroke subjects and control subjects suggest a higher common central drive during movement. Furthermore, results highlight the association between stroke-related alteration of intermuscular functional connectivity and the alteration of motor function.

## Introduction

Stroke leads to chronic motor deficits and involves impairment of voluntary motor skills consecutive to the central and peripheral alteration of neuromuscular function (Gracies, 2005). These changes include how the muscles are co-activated. Indeed, the connectivity between muscles is lower in stroke patients than in control subjects, which means that communication between a smaller number of muscles is involved in the realization of the patients’ movement (Houston et al., 2021, 2020). This strategy is all the more important when motor function is impaired (Houston et al., 2021, 2020) and could be responsible for the slowness and loss of smoothness of movement (Pierella et al., 2020). Conversely, movement is improved when the motor control strategies of patients are close to those of control subjects (Pierella et al., 2020). These observations are in agreement with the general consensus on impaired muscle co-activation after stroke, which in part involves the alteration of co-contraction in this population (Roh et al., 2013). Co-contraction is related to an involuntary contraction of both synergistic agonist and antagonist muscles (Gracies, 2005) that occurs in healthy subjects but which is excessive in stroke subjects (Angel, 1975) and can be partly responsible for the alteration of the motor function of the upper paretic limb (Chollet et al., 1991; Crafton et al., 2003; Ohn et al., 2013; Chalard et al., 2019). It should be noted that in this study, the synergistic muscles are all muscles involved in a movement. The agonist’s muscles develop an effort in the direction of the movement, and the antagonists develop an effort in the opposite direction.

The motor control mechanisms underlying the regulation of co-contraction between synergistic muscles can be approached according to the common drive theory proposed by De Luca and Erim (2002). By calculating the correlation between motor unit firing activities, the authors demonstrated a common activity between different motor units. Since the common frequency content of different muscles is associated with the coordination of the muscles in different tasks (Laine and Valero-Cuevas, 2017), there is a consensus in the literature that the statistical correlation between the electromyographic (EMG) signals of two synergistic muscles constituting a pair could reflect a common central drive involved in the control of the resulting motor action (De Luca and Erim, 2002; Boonstra, 2013). Thus, this common central drive is opposed to a more specific drive that could allow greater muscle selectivity. In other words, the higher the intermuscular coherence (IMC), the more common the central drive sent to the motor units of the synergistic muscles. IMC can be quantified in different frequency bands to reflect a common central descending drive that may contain a variety of information. Typically, IMC variations in the “alpha” (α) frequency band reflect a common central drive involved in posture-related muscle contractions or involuntary contractions (Kattla and Lowery, 2010; Boonstra, 2013). In the “gamma” (γ) frequency band, IMC modulation reflects a common central drive involved in elements including visual attention, motor planning, and cognition (Brown, 2000), while IMC modulation in the “beta” (β) frequency band is associated with a common central drive involved in voluntary contractions^15^. In control subjects, authors have demonstrated a link between IMC and changes in muscular synergies induced by different tasks, suggesting that IMC reflects a neural control strategy of functional coordination (Laine and Valero-Cuevas, 2017; Laine et al., 2021). In addition, the authors suggested that their results offer an interesting opportunity to explore and better understand the neural strategies of functional coordination in stroke subjects. In addition, it has already been shown in stroke subjects that α-IMC is lower in the paretic limb than in the dominant limb of control subjects when the studied muscle pair is composed of two muscles agonistic to the movement (Kisiel-Sajewicz et al., 2011). When the muscle pair is composed of an agonist muscle and an antagonist muscle, some authors observed lower IMC in the paretic limb of stroke compared to control subjects in both the α and β frequency bands (Yu et al., 2021). Conversely, a majority of studies have shown higher IMC in both the β and the γ band compared to control subjects (Kamper et al., 2014; Kitatani et al., 2016; Becker et al., 2018; Liu et al., 2022), suggesting a higher common drive directed to the synergistic muscles of stroke subjects. Furthermore, for the same muscle pair in stroke subjects, IMC appeared higher when the muscles acted as antagonists than when they acted as agonists (Kamper et al., 2014), unlike the results in control subjects (Hu et al., 2018). Such apparent contradictory results emphasize the importance of further studying IMC after stroke, especially according to the functional role of the muscles constituting the pairs of synergistic muscles involved in the movement.

To the best of our knowledge, no study has explored stroke-related changes in IMC compared with control subjects, including the paretic and non-paretic upper limbs, and accounting for the functional role of muscle pairs in an ecological task. In addition, no studies have investigated whether there is a relationship between IMC and antagonist co-contraction after stroke, even if Becker et al. (2018) suggested the involvement of IMC in antagonist co-contraction. Within the general aim of better understanding the motor control mechanisms involved in the impairment of motor function in stroke subjects, this study aimed to explore the effect of stroke on the common central drive sent to muscles involved in movement. Regarding hypotheses, given the literature evoked above on changes in IMC following stroke, we expected to observe a difference in IMC between the muscle pairs of the paretic limb among stroke subjects and muscle pairs of control subjects, as well as between the muscle pairs of both limbs of stroke subjects. Unlike control subjects, stroke subjects have different strategies for regulating IMC according to the functional role of muscle pairs (Kamper et al., 2014; Hu et al., 2018). Consequently, we expected to observe different IMC alterations between the antagonist-antagonist and the agonist-antagonist muscle pairs. For this purpose, the differences in IMC between the paretic and non-paretic upper limbs of stroke patients and the dominant limb of control subjects during elbow extension movements were investigated. This movement was chosen because it is an essential step in many daily life activities, the limitation of elbow extension movement being a frequent source of functional limitation. This analysis was performed in the β frequency band, given what these frequencies represent in the context of healthy and impaired motor control. This analysis was done for two pairs of muscles: one composed of two antagonist muscles and the other composed of an agonist muscle and an antagonist muscle.

## Materials and methods

### Participants

Twenty-four unilateral chronic stroke subjects (mean ± standard deviation of time after stroke: 37.56 ± 49.44 months) and twenty-four healthy control subjects were age-matched (see Table 1 for detailed demographics). The t-test performed between the age of the stroke and control groups did not reveal any significant differences (t(47) = 1.49 *p* = 0.14, ES = 0.42). For all subjects, the exclusion criteria were the presence of comprehension disorder, neurodegenerative disease, and pain in the upper limbs during active elbow extension. Patients had to be free of botulinum toxin A injections in the elbow flexors for at least four months. They also had to be able to actively extend the elbow by at least 20°.

**TABLE 1:** Detailed demographics and clinical of participants.

Subjects	Sex	Age (years)	Time since stroke (months)	EmNSA/64	Upper limb fugl Meyer/66	Stroke type	Stroke side, location
Controls (n = 24)	10M/14F	51 ± 14	/	/	/		

Stroke subjects	21M/4F	57 ± 13	38 ± 50	48 ± 14	40 ± 11		
1	M	61	51	27	38	Ischemic	Right, cortical and subcortical territories of MCA
2	M	59	18	60	46	Hemorrhagic	Right, subcortical territories of MCA
3	F	69	19	50	44	Ischemic	Right, Pons (paramedian)
4	M	65	75	50	32	Hemorrhagic	Left, basal ganglia, and internal capsule
5	M	50	30	60	42	Ischemic	Right, cortical and subcortical territories of MCA
6	M	57	14	61	45	Ischemic	Left, posterior limb of the internal capsule
7	M	75	26	56	26	Ischemic	Left, cortical, and subcortical territories of MCA
8	M	48	8	33	49	Ischemic	Left, cortical, and subcortical territories of MCA
9	M	65	116	54	30	Ischemic	Right, cortical and subcortical territories of MCA
10	M	49	13	62	53	Ischemic	Predominant right, Pons (paramedian)
11	F	33	9	63	45	Ischemic	Predominant left, Pons, and middle cerebellar peduncles
12	F	34	12	48	21	Ischemic	Left, subcortical territories of MCA
13	M	57	18	52	41	Ischemic	Right, cortical and subcortical territories of MCA
14	M	56	34	26	29	Ischemic	Right, cortical and subcortical territories of MCA
15	M	76	12	60	50	Ischemic	Left, subcortical territories of MCA and hippocampus uncus.
16	M	74	34	58	23	Ischemic	Right, Pons (paramedian)
17	M	67	6	63	47	Ischemic	Left, internal capsule
18	M	43	15	24	46	Ischemic	Right, cortical and subcortical territories of MCA
19	M	72	43	39	36	Hemorrhagic	Right, thalamus
20	M	41	15	41	58	Ischemic	Right, subcortical territories of MCA
21	M	52	36	64	41	Ischemic	Left, internal capsule
22	M	54	12	23	27	Hemorrhagic	Right, internal capsule and thalamus
23	F	39	52	64	63	Ischemic	Right, cortical and subcortical territories of MCA
24	M	54	27	35	47	Ischemic	Right, cortical and subcortical territories of MCA

The Erasmus modified Nottingham Sensory Assessment (EmNSA) is a somatosensory assessment in which 64 is the higher score. The upperlimb Fugl Meyer assessment is a sensorimotor assessment which 66 being the higher score.

Five stroke subjects and nine healthy control subjects were included in a study approved by the Research Ethics Committee of Toulouse University Hospitals (No. 07-0716). Eighteen stroke subjects were included in a routine care protocol (No. ID-RCB: 2017-A01616-47), and one stroke subject and fifteen healthy control subjects in an interventional protocol (No. ID-RCB: 2017-A01616-47). All these studies took place at the University Hospital of Toulouse and were conducted in accordance with the Declaration of Helsinki. All subjects provided written informed consent before study entry.

### Procedure

**FIGURE 1:**
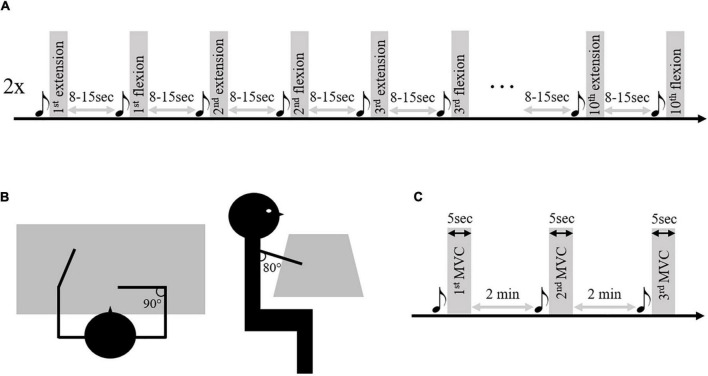
Illustration of the research protocol. Panel **(A)** represents the two series of 10 elbow extension/flexion movements performed by the patients and control subjects. Panel **(B)** represents the starting position. Panel **(C)** represents the maximal voluntary contraction (MVC) series performed on the isokinetic ergometer. The musical notes represent the sound signal indicating the beginning of the movement.

As presented in Delcamp et al. (2022), subjects sat in front of a table, arms resting at 80° of shoulder flexion, 90° of shoulder internal rotation, elbow flexed at 90°, and the torso secured at the chair (Figure 1). Following a sound signal, subjects were first asked to lift their arm from the table (up to 90° of shoulder flexion), followed by a maximal elbow extension movement at spontaneous speed, and finally to rest their arm now, extended back onto the table. At the next sound signal, subjects were asked to perform an elbow flexion movement following the same requirements. The sound signals were separated by a randomized rest period ranging from 8 to 15 seconds. Subjects performed a total of 20 elbow movements of extensions and flexions for each limb, divided into a randomized series of 10 movements (Figure 1). During these movements. After appropriate skin preparation according to SENIAM guidelines (Hermens et al., 2000), kinematic and electromyographic activity were recorded at a respective sample rate of 125 Hz (system OptiTrack; NaturalPoint Inc., Corvallis, OR, USA) and 1000 Hz (Biopac Systems Inc., Goleta, CA, USA; common mode rejection ratio (CMRR) > 110 dB; amplification gain: 1000). For the paretic and non-paretic limb of stroke subjects and the dominant limb of control subjects, EMG data were recorded from the long head of the triceps brachii (TB) as the main elbow extensor muscle and from the short and long head of the biceps brachii (BB) and brachioradialis (BR) as the main elbow flexor muscles. In this study, we exclusively analyze elbow extension movements because this movement presents a significant functional limitation for stroke subjects.

### Data analysis

#### Pre-processing

**FIGURE 2:**
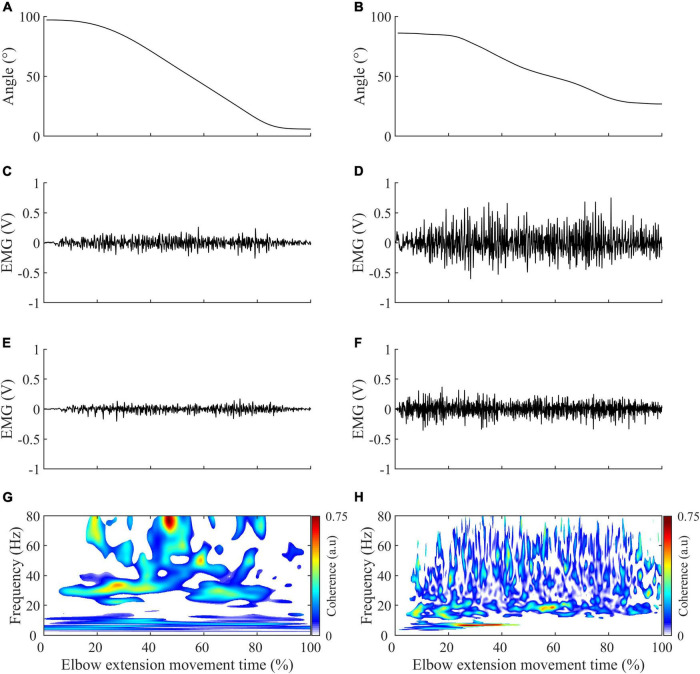
Illustration of the typical recording obtained in control (left panels) and stroke subjects (right panels) during elbow extension. **(A,B)** Elbow range of motion variation. **(C,D)** Triceps brachii electromyographic activity in volt (V), with a gain setting of 1000. **(E,F)** Brachioradialis electromyographic activity in volt (V), with a gain setting of 1000. **(G,H)** Significant intermuscular coherence map calculated from the triceps brachii and brachioradialis muscle activity represented in the second and third row and according to the methodology described in methods.

Kinematic continuous data were low-pass filtered at 6 Hz (Cahouët et al., 2002; Delcamp et al., 2022). The onset and offset of each elbow extension movement were detected with a threshold of 0.01°/s applied to the elbow’s instantaneous angular velocity (Chalard et al., 2019). Electromyographic continuous data were band-stop filtered at 49–51 Hz (Krauth et al., 2019) to remove power line noise. As was done in Fauvet et al. (2019, 2021), Glories et al. (2021), and Delcamp et al. (2022), the EMG signal was then 3-100 Hz band-pass filtered to keep the denoised part of the EMG signal energy that is necessary for reliable quantification of intermuscular coherence in the frequency band of interest in the present study. All the filters were fourth-order, zero-lag Butterworth types. Typical recordings of kinematics and electromyographic activity of the triceps brachii and brachioradialis for healthy and control subjects are represented in Figure 2.

#### Data processing

Spastic co-contraction, here more appropriately called antagonist co-contraction, was calculated as the average of the elbow’s flexors (BB & BR) electromyographic root mean square value during elbow extension, normalized by their average root mean square value during maximal isometric voluntary elbow flexion contraction (Chalard et al., 2019). This maximal voluntary contraction was obtained from a series of three five-second voluntary maximal contractions, two minutes apart, performed on an isokinetic ergometer with shoulder flexion of 80°, an internal rotation of 90°and the bust attached. The EMG of the maximal contraction selected to calculate the antagonist co-contraction was that of the contraction (among the 3) where the torque was maximal.

**FIGURE 3:**
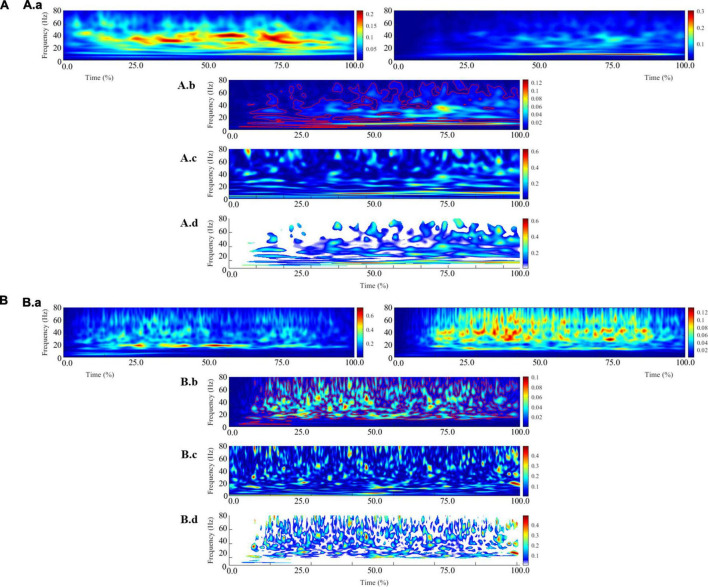
Illustration of the different steps involved in calculating intermuscular coherence for a typical control subject **(A)** and a typical stroke subject **(B)**. The **(A.a, B.a)** panels represent the average of the auto-spectrum calculated for each movement from the biceps brachii (left) and brachioradialis (right) electromyographic signals. These panels represent the frequential properties of the respective muscular signals. The panels **(A.b, B.b)** panels represent the cross-spectrum, and the red contours identify areas in the time-frequency plane where correlations between the electromyographic signals are significant. The **(A.c, B.c)** panels represent the wavelet magnitude-squared coherence between the two electromyographic time series. The **(A.d, B.d)** panels represent the wavelet magnitude-squared coherence where the correlation between the EMG signals is significant.

For IMC analysis, the continuous EMG data were segmented from 3 s before and 3 s after each movement to limit the alteration of the ends of the signals during the pre-processing. Then the signals were normalized to account for inter-movement time variability (Fauvet et al., 2019). During this step, the 3 s of retained signals at the movement’s ends were removed. For each active limb of each subject, IMC was calculated in the time-frequency domain between the TB-BR electromyographic signals (as an agonist/antagonist muscle pair) and the BB-BR electromyographic signals (as an antagonist-antagonist muscle pair). It is noteworthy that, within the debate on EMG rectification from coherence analysis, the EMG signals were not rectified to both satisfy theoretical arguments regarding the calculation of coherence and to avoid subsequent inconsistencies in power and IMC coherence spectra estimates (Bigot et al., 2011; McClelland et al., 2012). The parameters “nvoice,” “J1,” and ‘wavenumber,” which represent the scale resolution of wavelets, the number of scales, and the Morlet mother wavelet parameter, were respectively set to 7, 50, and 10 to yield accurate identification of oscillatory activity in the 0.23 Hz to 79.97 Hz frequency range in 0.23 steps (Figure 3). These parameters set the time-frequency precision compromise to a 0.1 s–3 Hz precision window within the β (13–31 Hz) frequency band. IMC was calculated by normalizing the cross-spectrum by the product of the auto-spectrum as follows:


$$R_{E\,M\,G\,1/E\,M\,G\,2}^{2}\,\left(\omega ,u\right)=\frac{{\left|S_{E\,M\,G\,1/E\,M\,G\,2}\,\left(\omega ,u\right)\right|}^{2}}{S_{E\,M\,G\,1}\,\left(\omega ,u\right)\,S_{E\,M\,G\,2}\,{\left(\omega ,u\right)}^{{}^{\prime }}}$$

where EMG1 and EMG2 are EMG time series, S_*EMG*1/EMG2_ (ω,u) is the wavelet cross-spectrum between EMG time series at frequency ω and time u; SEMG1 (ω, u) and SEMG2 (ω, u) are wavelet auto-spectra of EMG time series at frequency ω and time u. Then, IMC was quantified in the β frequency band and in a time window of 200 ms before maximal peak velocity as the volume under the IMC values, which were previously detected as significant (Figure 3).

#### Statistical analysis

Independent sample Student’s t-test was performed to compare the antagonist co-contraction between the paretic limb of stroke subjects and the dominant limb of control subjects.

In order to control the possible effect of angular velocity, elbow angle (Duclay et al., 2009; Becker et al., 2018), and age of the subject (Jaiser et al., 2016), as well as to consider differences in antagonist co-contractions (Houston et al., 2021), ANCOVAs were performed on IMC values with four covariables (mean elbow angle and mean angular velocity on the IMC quantification window, age, and antagonist co-contractions):

- One between the paretic limb of stroke subjects and the dominant limb of control subjects

- One between the paretic and non-paretic limbs of stroke subjects

- One between the non-paretic limb of stroke subjects and the dominant limb of control subjects.

Since the Shapiro-Wilk test indicated non-normality of the residuals and the ANCOVA with a balanced design is the most robust and powerful statistical analysis available (Rheinheimer and Penfield, 2001), BoxCox transformations of IMC data were applied.

To study a possible link between IMC and motor function and to consider the non-normality of IMC values, a partial Spearman correlation was performed for the paretic limb of all the stroke subjects between the IMC of the antagonist muscle pair and the antagonist co-contraction (as two dependent variables) by controlling potential covariables: angular velocity, elbow angle (Duclay et al., 2009; Becker et al., 2018), and age of the subject (Jaiser et al., 2016). This correlation was done with and without outliers (± two residuals standard error).

For the ANCOVAs, significance was corrected at *p* < 0.017. For the partial Spearman correlation, significance was set at *p* < 0.05.

## Results

**FIGURE 4:**
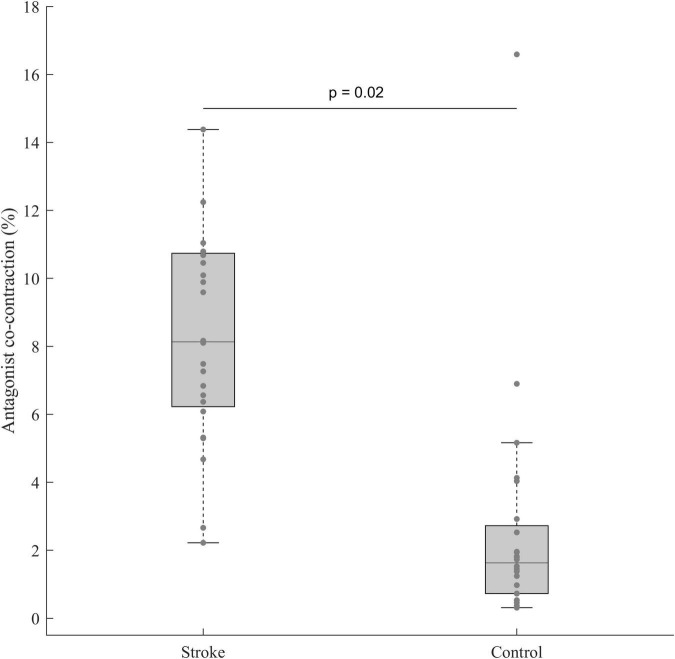
Antagonist co-contraction in the paretic limb of stroke subjects (n = 24) and the dominant limb of control subjects (*n* = 24). In each box, the center line represents the median, the top, and bottom of the box correspond to the 25th and 75th percentiles, and the whiskers represent the 10th and 90th percentiles.

The student t-test performed between the paretic limb of stroke subjects and the dominant one of control subjects revealed a higher antagonist co-contraction for stroke subjects with a moderate effect size (t(46) = 2.38, *p* = 0.02, *d* = 0.69, 95% CI = [0.08:1.29]) (Figure 4).

**FIGURE 5:**
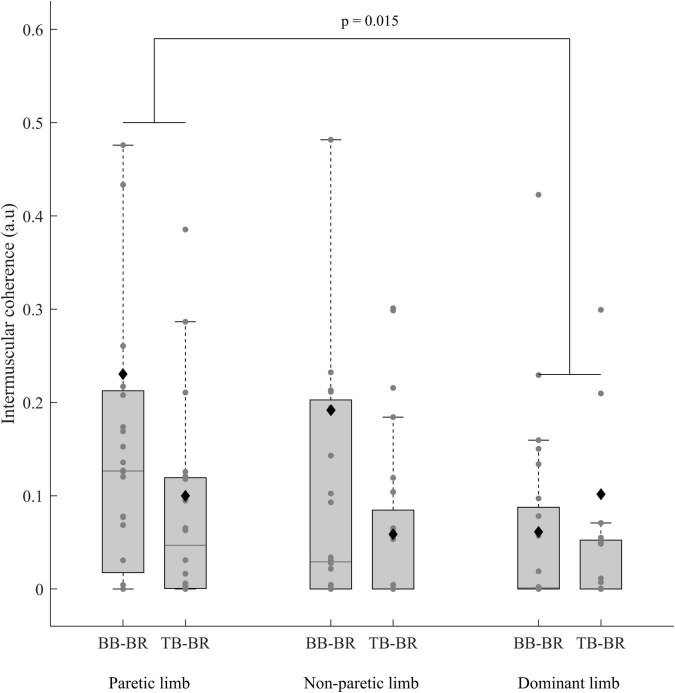
IMC (before BoxCox transformation) of the paretic and non-paretic limbs of stroke subjects (*n* = 24) and the dominant limb of control subjects (n = 24) for both the antagonist-antagonist and agonist-antagonist muscle pairs (Biceps brachii-Brachioradialis (BB-BR) and Triceps brachii-Brachioradialis (TB-BR)). The *p*-values shown in the figure are for the effect of a limb in the ANCOVA. In each box, the center line represents the median, the top, and bottom of the box correspond to the 25th and 75th percentiles, and the whiskers represent the 10th and 90th percentiles. The diamond markers represent the mean of each group.

**FIGURE 6:**
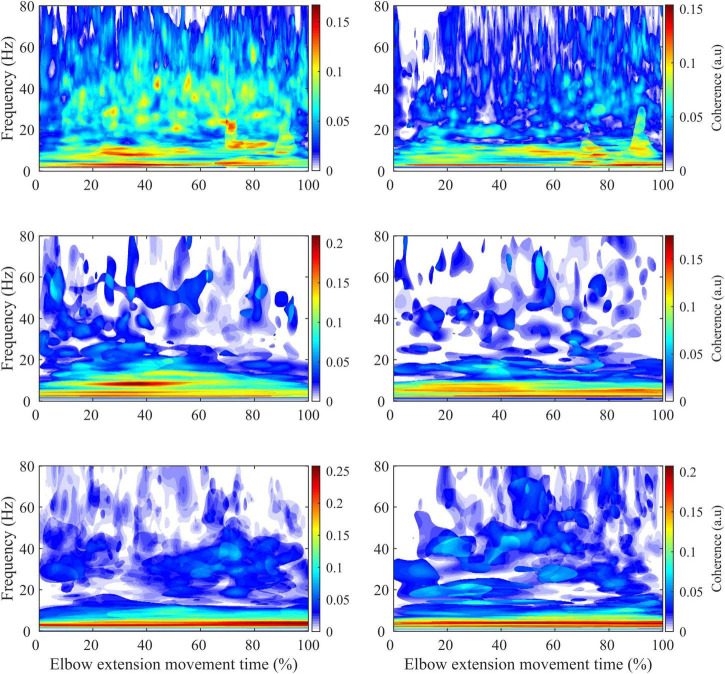
Average intermuscular coherence map for the biceps brachii-brachioradialis **(left)** and triceps brachii-brachioradialis **(right)** muscle pairs of the paretic limb (first row) and non-paretic limb of the stroke subjects (second row) and the dominant limb of the control subjects (third row).

The ANCOVAs showed a significant limb effect between the paretic limb of stroke subjects and the dominant limb of control subjects (*p* = 0.015) and a muscle pair effect only for the paretic and non-paretic limbs of stroke subjects (*p* = 0.016) (Table 2 and Figures 5, 6).

**TABLE 2:** Results of ANCOVAs performed between the paretic limb of stroke subjects and the dominant limb of control subjects (A), the paretic and the non-paretic limbs of stroke subjects (B) and the non-paretic limb of stroke subjects, and the dominant limb of control subjects (C). There were a limb factor and a muscle pair factor (BB-BR et TB-BR) with four covariables: Velocity (average velocity of the IMC quantification window), Angle (average angle of the IMC quantification window), Age of subjects, and Antagonist co-contraction.

A. Paretic vs. Dominant limbs	F	p	η^2^p
Limb	5.76	0.015*	0.06
Muscle pair	2.56	0.113	0.03
Limb * Muscle	1.06	0.305	0.01
Velocity	1.25	0.267	0.01
Angle	1.67	0.200	0.02
Age	1.92	0.801	0.01
Antagonist co-contraction	0.00	0.990	0.00
**B. Paretic vs. Non-paretic limbs**			
Limb	2.12	0.148	0.02
Muscle pair	5.91	0.016*	0.63
Limb * Muscle	0.01	0.902	0.00
Velocity	0.00	0.963	0.00
Angle	0.11	0.736	0.00
Age	0.08	0.779	0.00
Antagonist co-contraction	0.12	0.725	0.00
**C. Non-paretic vs. Dominant limbs**			
Limb	0.38	0.537	0.00
Muscle pair	2.44	0.122	0.03
Limb * Muscle	0.64	0.425	0.00
Velocity	1.85	0.177	0.02
Angle	1.63	0.206	0.02
Age	0.04	0.839	0.00
Antagonist co-contraction	6.40	0.013	0.06

The symbol * represents a significant effect of ANCOVA with a *p* corrected to *p* < 0.017.

**FIGURE 7:**
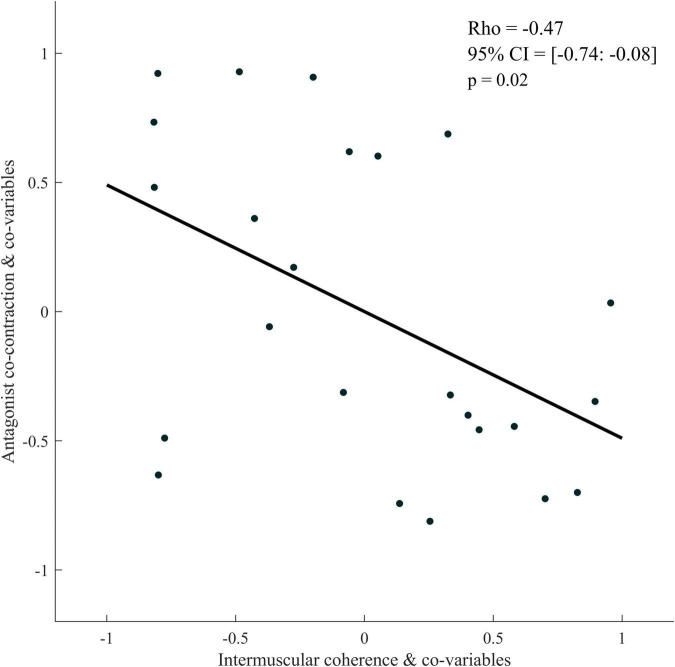
Partial rank spearman correlation plot between antagonist co-contraction and intermuscular coherence of the Bicep Brachii-Brachioradialis muscle pair for the paretic limb of stroke patients. The data plotted are the residuals of the spearman rank correlation performed between antagonist co-contraction and the covariables and corticomuscular coherence and the covariables.

The partial Spearman correlations showed a significant negative relationship between antagonist co-contraction and IMC_BB–BR_ (respectively, with and without an outlier: Rho = −0.47, 95% CI = [−0.74: −0.08], *p* = 0.02; Rho = −0.47, 95% CI = [−0.79: −0.01], *p* = 0.04) (Figure 7).

## Discussion

This study aimed to assess the differences in IMC between stroke and control subjects in both a muscle pair composed of antagonist muscles (BB-BR) and a muscle pair composed of an agonist muscle and an antagonist muscle (TB-BR). This approach allowed us to consider the functional role (i.e., agonist or antagonist) of the synergistic muscles constituting the pairs implicated in changes in intermuscular functional connectivity after stroke.

First, our results showed higher antagonist co-contraction for the paretic limb of stroke than for control subjects. This result fully agrees with the literature (Gracies, 2005; Delcamp et al., 2022) and highlights the impairment of motor function present in the population of stroke subjects included in this study.

Concomitantly, our key results showed a significantly higher IMC when calculated from muscle pairs of the paretic limb of stroke subjects compared with the muscle pairs of the dominant limb of control subjects. In addition, our results showed significantly higher IMC for the BB-BR muscle pair compared to the TB-BR, only for stroke patients. Finally, a negative linear link between the IMC of the BB-BR muscle pair and antagonist co-contraction of the paretic limb of stroke subjects were highlighted.

### Alteration of IMC for the paretic limb of stroke subjects

While controlling for possible methodological biases and differences in motor function between subjects, we found that IMC calculated from the muscles of stroke subjects’ paretic limb was higher when compared to the dominant limb of control subjects. This result agrees with our hypotheses, as we expected to observe a difference in motor control between the paretic limb of stroke subjects and control subjects. These results are also in agreement with most of the literature studying the differences in IMC between stroke and control subjects for muscle pairs composed of an agonist and an antagonist’s muscles, where IMC appears higher for muscles of stroke subjects (Kamper et al., 2014; Becker et al., 2018). However, even though the non-paretic limb of the patients is not healthy, the ANCOVAs performed did not show any difference in IMC between the paretic and non-paretic limbs of the patients, which is in agreement with the work of Kitatani et al. (2016). Furthermore, work performed based on corticomuscular coherence analysis (between the brain and muscles) has also demonstrated the effect of stroke on the nature of the central drive involved in motor control for the paretic limb (Krauth et al., 2019; Delcamp et al., 2022). Therefore, our results agree with these studies concerning the alteration of the motor control of stroke subjects’ paretic limbs.

Different hypotheses could explain the effect of stroke on the IMC of the paretic limb. First, it is interesting to discuss the observed IMC differences concerning De Luca and Erim’s common drive theory (De Luca and Erim, 2002). These authors argue that higher IMC would reflect a greater common central drive sent to the motor units of synergistic muscles. Given our results, we can assume that the unilateral central lesion would thus lead to an alteration in the programming of the central drive, which might be more common concerning muscles of the paretic limb. Conversely, this could also represent an effect of stroke on the decreased muscle selectivity of the paretic limb since the muscles (which have the same or opposite functional role) would receive less selective control than those of the non-paretic limb or control subjects. Thus, how the central nervous system drives synergistic muscles appears to be modified for the muscles of the paretic limb of stroke subjects compared to the muscles of control subjects. The development of new muscular synergies seems to occur for the paretic limbs of these stroke subjects, which would modify how the muscles are coordinated during movements.

Secondly, these findings can also be discussed in relation to the alteration of spinal mechanisms after stroke. Indeed, spinal regulatory mechanisms can contribute to modifying IMC because they regulate efferences and differences since, in stroke subjects, reciprocal (Delwaide and Oliver, 1988; Nakashima et al., 1989) and recurrent inhibition mechanisms are decreased (Mazzocchio, 1997). A decrease in spinal inhibition mechanisms could therefore be at the origin of a less regulated central descending drive and, therefore, of a higher IMC calculated from the muscles of the paretic limb than that calculated in control subjects. In contrast to the elements discussed above, we can assume that the initial central drive could be similar to the non-paretic limb of stroke or control subjects, but its spinal regulation would be different.

The changes in IMC observed in stroke patients could reflect the mechanisms underlying the regulation of muscular synergistic co-activation and their alteration, resulting in a modification of antagonist-agonist co-contraction. IMC appears to be higher for the antagonist-antagonist muscle pair (BB-BR) compared to the agonist-antagonist muscle pair (TB-BR) only for stroke subjects. A more common drive directed to muscles with the same functional role could be explained by a “simple, functional control scheme to compensate for lower efficiency in motor execution” (Houston et al., 2020), which could lead to less muscular selectivity toward the antagonist’s muscles. Thus, muscles with the same functional role would be controlled as an entire entity by a common central descending drive less selective than the drive for TB-BR (De Luca and Erim, 2002). Given the difference we observed between these two muscle pairs, we can suggest a more general way to alter the motor network interactions in stroke patients, in line with recent studies by Houston et al. (2020, 2021). In this sense, in stroke patients, the elbow flexors may represent a group of functional units (Reyes et al., 2017) receiving a more common central drive than the agonist-antagonist pair.

### Functional simplification of motor control after stroke

Our results indicate for the first time a negative relationship between antagonist co-contraction and IMC of the antagonist-antagonist muscle pair (BB-BR) in the paretic limb of stroke subjects during active elbow extension. This finding agrees with that of Liu et al. (2022) (Liu et al., 2022), who reported that the IMC of antagonist-antagonist and agonist-agonist muscle pairs are positively correlated to motor function performance in stroke patients, which suggests the involvement of intermuscular functional connectivity and, by extension, of motor network interactions— in the regulation of motor function. While stroke patients have higher IMC than control subjects (Kamper et al., 2014; Kitatani et al., 2016; Becker et al., 2018), this correlation obtained in the current study further supports the proposition that stroke patients with higher IMC would exhibit lower antagonist co-contraction. Although it may seem at first to be counterintuitive that a decrease in antagonist co-contraction – known to contribute to the improvement of motor function – would be associated with an increase in IMC – though as an alteration of motor control –, our results conversely suggest that increased common central drive to antagonist’s muscles would allow limiting the amount of their exaggerated co-contraction in stroke subjects. The observed changes in IMC would therefore reflect a functional simplification of intermuscular connectivity rather than a maladaptive mechanism. This reinforces the conclusion of the previous section and, in line with Houston et al. (2021), suggests that a completely new reorganization of motor network interactions occurs after a stroke, which may allow patients to accommodate the general alteration of central motor control.

## Conclusion

The IMC differences between the stroke subjects’ paretic limb and the dominant limb of control subjects reinforced the results of the literature about the effects of stroke on intermuscular connectivity and completed it with an analysis of pairs of muscles with the same and opposite functional roles. Moreover, the negative correlation in stroke subjects’ paretic limbs between antagonist co-contractions of these muscles and their IMC could highlight a functional simplification of motor control in this population. Finally, the IMC appeared to be higher for muscles with the same functional role (BB-BR) for stroke patients, corroborating this hypothesis of simplifying motor control.

## Limits

Conclusions on IMC should be treated with caution given the possible influence of velocity and muscle length on IMC values (Jesunathadas et al., 2013; Becker et al., 2018), although we have statistically controlled these covariables. Surface electromyographic crosstalk can also be a potential bias. However, to date, there is no method to remove this crosstalk following an electromyographic recording with Ag-AgCl bipolar electrodes without altering the intermuscular coherence. In the present study, particular attention was given to EMG placement. We considered the crosstalk equivalent between the two groups, making comparisons possible, as was done in previous studies on IMC (Charissou et al., 2017; Laine et al., 2021). Although stroke subjects present an alteration of motor function, it is relevant to note that the locations of their brain lesions varied. Although no study to date has shown an effect of lesion location on patient IMC, we performed hierarchical clustering to explore a possible grouping of patients’ paretic limb IMC (BB-BR and TB-BR), which did not show a hierarchical link related to the lesion (size and location). However, we cannot exclude that these parameters may influence stroke subjects’ IMC. Finally, it is also important to note that the muscle mass of the subjects was previously associated with their IMC in elderly people (Nojima et al., 2020) and stroke subjects (Wang et al., 2015) and that this parameter could influence the differences in IMC between limbs.

## Data availability statement

All the data that support the findings of this study are available from the principal investigator DG and the scripts are available from the corresponding author, DA, upon request. Requests to access these datasets should be directed to DA.

## Ethics statement

The studies involving human participants were reviewed and approved by Protection of Persons Committee (Tours, Ouest 1): No. ID-RCB: 2017-A01616-47; Research Ethics Committee of Toulouse University Hospitals: No. 07-0716; Protection of Persons Committee (Sud-Est 1): No. ID-RCB: 2017-A01616-47. The patients/participants provided their written informed consent to participate in this study.

## Author contributions

CD: conceptualization, methodology, software, formal analysis, investigation, data curation, and writing (original draft and visualization). CC: resources, writing (review and editing). AC: investigation, software, and writing (review and editing). DA: methodology, software, and writing (review and editing). DG: software, resources, writing (review and editing), and project administration. All authors contributed to the article and approved the submitted version.
